# Pharmacokinetics and Toxicity Evaluation of a PLGA and Chitosan-Based Micro-Implant for Sustained Release of Methotrexate in Rabbit Vitreous

**DOI:** 10.3390/pharmaceutics13081227

**Published:** 2021-08-09

**Authors:** Soumyarwit Manna, Anna M. Donnell, Rafaela Q. Caixeta Faraj, Blanca I. Riemann, Christopher D. Riemann, James J. Augsburger, Zelia M. Correa, Rupak K. Banerjee

**Affiliations:** 1 Department of Mechanical and Materials Engineering, University of Cincinnati, Cincinnati, OH 45221, USA; soumyarwit_m@hotmail.com; 2 Department of Chemistry, University of Cincinnati, Cincinnati, OH 45221, USA; donnelaa@ucmail.uc.edu; 3 Department of Ophthalmology, University of Cincinnati, Cincinnati, OH 45221, USA; rafaqcaixeta@hotmail.com (R.Q.C.F.); briemann@fuse.net (B.I.R.); criemann@cincinnatieye.com (C.D.R.); james.augsburger@uc.edu (J.J.A.); correazm@gmail.com (Z.M.C.); 4 Department of Ophthalmology, Bascom Palmer Eye Institute, University of Miami, Miami, FL 33146, USA

**Keywords:** methotrexate, chitosan, PLA/PLGA, sustained release, micro-implant, animal model, minimally invasive

## Abstract

The present research investigates the pharmacokinetics and toxicity of a chitosan (CS) and poly(lactic-co-glycolic) acid (PLGA)-based methotrexate (MTX) intravitreal micro-implant in normal rabbit eyes. PLGA and CS-based micro-implants containing 400 µg of MTX were surgically inserted in the vitreous of twenty-four New Zealand rabbits using minimally invasive procedures. The PLGA-coated CS-MTX micro-implant and the placebo micro-implant were inserted in the right eye and in the left eye, respectively, of each rabbit. The intravitreal MTX concentration was evaluated on Days 1, 3, 7, 14, 28 and 56. A therapeutic concentration of MTX (0.1–1.0 µM) in the rabbit vitreous was observed for 56 days. The release of MTX in the therapeutic release phase followed first-order kinetics. Histopathologic evaluation on Days 14, 28 and 56 of the enucleated eyes demonstrated no signs of toxicity or any anatomical irregularity in the vitreoretinal domain. Additionally, the micro-implants were stationary at the position of their implantation throughout the duration of the study. The PLGA-coated CS-MTX micro-implant can serve as a potential alternative to the current treatment modality of intravitreal MTX injections based on its performance, thereby avoiding associated complications and the treatment burden of multiple injections.

## 1. Introduction

Methotrexate (MTX), an antimetabolite chemotherapeutic drug, has gained a substantial reputation in treating various types of cancer and autoimmune diseases [1]. In the domain of ophthalmology, intravitreal injections of MTX have been administered to treat various vitreoretinal (VR) diseases such as: (1) primary central nervous system lymphoma (PCNSL) with intraocular (vitreoretinal) involvement [2,3,4], also known as primary intraocular lymphoma (PIOL); (2) severe and recalcitrant intraocular inflammation (uveitis) [5,6,7,8]; and (3) certain cases of rhegmatogenous retinal detachment associated with elevated risk for proliferative vitreoretinopathy (PVR) [9,10]. Furthermore, in clinical practice, there has been a gradual incorporation of the off-label use of this drug, as described hereafter.

Various administration regimens have been employed when treating with intravitreal MTX injection. A representative dosing regimen would be either two intravitreal injections every week until apparent clinical improvement is demonstrated or one intravitreal injection every week thereafter until complete clinical regression in the eye(s) is observed. Alternatively, one intravitreal injection every month for at least three additional months is administered. Typically, this regimen accumulates to 20 to 25 injections or more [11]. A single intravitreal injection of MTX at the 200 to 400 µg/0.1 mL dose has shown good tolerance. Nevertheless, all intravitreal injections bear a potential risk of exogenous endophthalmitis, induced rhegmatogenous retinal detachment and elevated intraocular pressure [12]. The cumulative risk of these potential side effects and complications combined with the inconvenience and discomfort of multiple invasive procedures cannot be ignored when multiple intravitreal MTX injections are administered. Several cases of corneal epithelial toxicity [13], induced retinal cotton wool spots and macular edema [14] have been reported even at a dose of 400 µg/0.1 mL.

The evaluation of in vitro cytotoxic activity of MTX on 63 different cell lines showed the therapeutic levels of MTX range from 0.1 μM to 1 μΜ, with a mean IC_50_ of 0.32 μM [15]. Accordingly, a concentration range of 0.1–1 µM of MTX is considered to be therapeutic. When administered by intravitreal injection, the hydrophilic MTX drug (log p = −1.85) undergoes rapid clearance in the intravitreal domain (half-life of 14.3 h) [15]. Previously intravitreal MTX concentrations of 0.1–1 µM from one injection have been observed to have therapeutic effectiveness for a limited duration of 48–72 h [15,16]. It is expected that a prolonged therapeutic efficacy can be attained by delivering MTX through a sustained-release intravitreal drug delivery device (micro-implant) in the intravitreal domain, without causing any collateral complications. Currently, there are several FDA-approved intravitreal micro-implants, which deliver antiviral medications and corticosteroids [17], which are relatively hydrophobic in nature. These FDA-approved intravitreal implants along with other intravitreal drug delivery systems have been based on lipophilic (or hydrophobic) biodegradable polymers such as lactic and glycolic acid-based matrices such as poly-lactic acid (PLA), poly-glycolic acid (PGA), their copolymers and derivatives poly(lactic-co-glycolic) acid (PLGA) [18,19,20]. The issue with the existing polymer matrices (PLA, PGA and PLGA) is that they are lipophilic in nature and do not blend well with hydrophilic drugs such as MTX. Another disadvantage of these lipophilic polymer matrices is that they degrade very slowly even after the drug has been released, causing local toxicity [21]. At present, there is no sustained drug delivery platform for hydrophilic drugs, such as MTX, to treat VR diseases in commercial practice, which poses major challenges to develop a satisfactory micro-implant device.

Chitosan (CS) was chosen as the polymer matrix to fabricate the micro-implant, as it has been previously used as a delivery vehicle for MTX (hydrophilic drug) in other drug delivery formulations due to its similar hydrophilic nature [22,23,24,25,26]. CS is a copolymer of N-acetylglucosamine and glucosamine, which is a fully or partially N-deacetylated (DA) derivative of the natural polymer chitin. CS has been recognized as a Generally Recognized as Safe (GRAS) material by the FDA [27]. It has been reported by de la Fuente et al. [27] that CS-based ocular drug delivery formulations have shown good tolerance and acceptability in terms of toxicity. However, to restrict the rapid release of the hydrophilic MTX from the hydrophilic CS matrix, a lipophilic coating was imperative. Because PLA and PLGA have been extensively used in FDA-approved intraocular implants [17], they were chosen as the coating polymer to the hydrophilic CS-MTX matrix.

Our group developed a biodegradable intravitreal micro-implant platform that delivers a sustained therapeutic dose of MTX to the VR domain for several months [11,28,29,30,31,32]. Initially, a CS and PLA-based MTX intravitreal micro-implant device was developed that was able to maintain a sustained release of MTX between 0.2 and 2 µg/day in vitro for more than 50 days [29] and a therapeutic concentration of 0.1–1 µM MTX for ~30 days in vivo [11]. Non-invasive methodologies such as electroretinogram (ERG) [28] and B-scan ultrasound (US), as well as histopathology [30], have suggested favorable safety and toxicity of this micro-implant in normal rabbit eyes.

In a subsequent study, a PLGA and CS-based MTX intravitreal micro-implant was developed, which demonstrated an improved sustained MTX release rate of 0.2–2 µg/day for ~3–5 months in vitro [32]. In this in vitro study, the influence of (a) molecular weight of the PLA/PLGA and (b) the composition of the PLGA polymer on the biodegradation characteristics of the micro-implant, including swelling and disintegration of the micro-implant, was analyzed. The molecular weight of CS used was 50,000–190,000 with a degree of deacetylation (DA%) ≥ 75%. (Sigma Aldrich, St. Louis, MO, USA). The evaluated molecular weight of ester-terminated PLGA 5050 (PLA/PGA copolymer ratio in PLGA is 50:50) (Lactel Biodegradable Polymers, AL) with an inherent viscosity of 0.82 in hexafluoroisopropanol at 30 °C was Mw = 287,000 with a polydispersity index of 2.78 [32]. The safety and toxicity of this improved PLGA and CS-based MTX micro-implant were validated in normal rabbit eyes using *non-invasive* techniques such as ERG, US, slit-lamp biomicroscopy (SLB), fundoscopy and intraocular pressure (IOP) measurement for a period of approximately 2 months [31]. Non-invasive evaluation proved the micro-implant to be safe and well tolerated in rabbit eyes for the whole duration of the study. In the same pre-clinical trial, we evaluated the pharmacokinetics and gross morphology of the micro-implant concurrently using minimally invasive techniques for improved knowledge of the safety of the micro-implant in the vitreoretinal domain.

In our pilot in vivo studies [11,28,30], the PLA-coated CS-MTX micro-implant had a different composition of the coating polymer, which resulted in a shorter duration of release (~1 month in vivo). Additionally, the scope of the pilot studies was limited due to the limited number of rabbits (n = 8). Subsequently, a recent study involving the improved PLGA-coated CS-MTX micro-implant was focused on non-invasive parameters only (ERG, US, SLB, fundoscopy and IOP) [31]. This study is novel because it presents findings on the invasive parameters, such as pharmacokinetics and toxicity evaluation of the PLGA-coated CS-MTX micro-implant, in thirty rabbit eyes. Additionally, this improved micro-implant design resulted in a significant increase in the release duration (~2 months in vivo), as presented hereafter.

## 2. Materials and Methods

**Figure 1:**
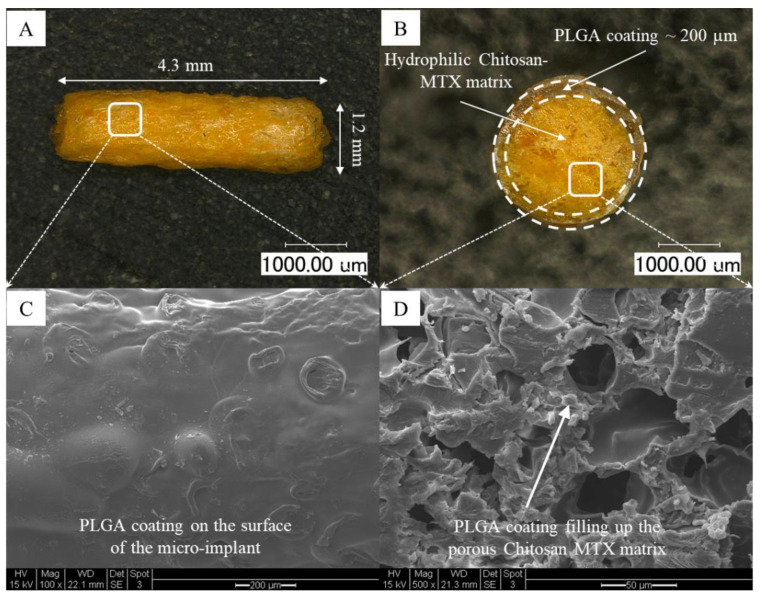
Optical microscopy images of the PLGA-coated CS-MTX micro-implant showing the top view (**A**) and the cross-sectional view (**B**). The microstructure of the micro-implant can be observed in the SEM images showing the top view (**C**) and the cross-sectional view (**D**).

*Fabrication of the Micro-implant:* In this in vivo study, the PLGA 5050 (PLA/PGA ratio = 50:50)-coated CS-MTX micro-implant was used. It was fabricated based on the method described in our earlier study [29,32]. Briefly, CS-MTX fibers were obtained by freeze-drying the mixture of CS and MTX in 0.1 N HCl in Tygon^®^ tubing (Saint-Gobain, Malvern, PA, USA) of a 1/16-inch internal diameter. Subsequently, these fibers were cut to desired lengths under a microscope, and then a ~200 µm coating of PLGA 5050 was applied on the surface using dip-coating techniques. As described earlier [29,32], the microstructure and the morphology of the micro-implant were evaluated using optical microscopy (Keyence Digital Microscope, VHX-600, Osaka, Japan) and scanning electron microscopy (SEM) (FEI XL 30-FEG, FEI, Hillsboro, OR, USA) using an accelerating voltage of 15 KV. The morphology and microstructure of the PLGA-coated CS-MTX micro-implant are shown in Figure 1.

The final dimensions of the PLGA-coated CS-MTX micro-implant containing 40% *w/w* MTX were ~4.3 mm in length and ~1.2 mm in diameter. The weight of the micro-implant containing 40% *w/w* MTX was ~1 mg, which translated to a drug loading of ~400 µg of MTX. The placebo micro-implant was fabricated following the same procedure without the drug. The rationale behind using the 40% *w*/*w* MTX loading was to compare the same dosage of MTX (400 µg of MTX), as administered in an intravitreal injection used in clinical practice. The micro-implants were sterilized under UV radiation for 20 min prior to surgical procedures.

*Design of the Non-invasive* In Vivo *Experiment*: Thirty immune-competent New Zealand albino rabbits, each weighing 2–3 kg, were used in this study. A sterilized MTX-loaded micro-implant and a sterilized placebo micro-implant (without MTX) were surgically implanted in the right eye and in the left eye, respectively, as per the study plan shown in Table 1. All procedures for the rabbit surgery were in accordance with the Institutional Animal Care and Use Committee protocol (IACUC No. 12-09-13-01, University of Cincinnati, dated: 21 November, 2012) and followed the ARVO Statement for the Use of Animals in Ophthalmic and Vision Research.

**Figure 2:**
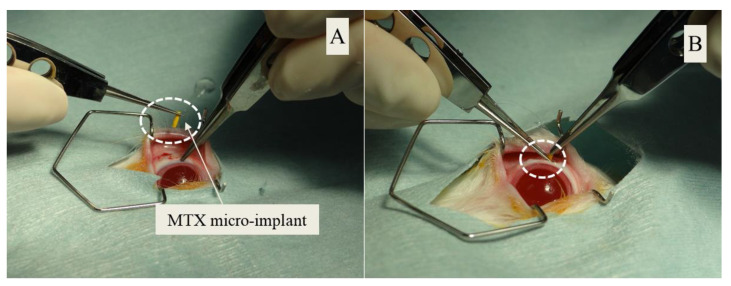
(**A**) MTX micro-implant before surgical insertion; (**B**) surgical insertion of the MTX micro-implant.

*Surgery*: The details of the surgery for this study are reported in our recent study [31] involving the non-invasive evaluation of safety and toxicity of the same micro-implant. Briefly, each animal was evaluated for the vital signs (heart rate, respiration rate, temperature) prior to surgery (PS) and verified to be within normal limits. Each rabbit was anesthetized with a mixture of xylazine hydrochloride (5 mg/kg) and ketamine hydrochloride (45 mg/kg) by intramuscular injection. Buprenorphine hydrochloride (0.02 mg/kg (Sub-Q)) was administered as an analgesic, and glycopyrrolate (0.006 mg/kg (IM)) was administered to stabilize the heart rate. For further supplementation of the anesthesia, tetracaine hydrochloride (0.05%) eye drops were administered topically to the eyes. The body temperature of the rabbit was maintained at 37 °C. An eyelid speculum was inserted to expose the right eye. A full-thickness eye wall incision was carried out parallel to the limbus at a distance measured 6 mm from the limbus in the superotemporal quadrant using a 1.1 mm side port paracentesis surgical knife. Subsequently, a PLGA-coated CS-MTX micro-implant was inserted through the eye wall incision using McPherson forceps (Figure 2). The conjunctival–scleral wound was immediately closed using a full-thickness mattress suture of 7-0 polyglactin 910 (suture material: Ethicon, Cincinnati, OH, USA). Then, the same procedure was repeated on the left eye except for the insertion of a placebo micro-implant (without MTX loading) in that eye. Immediately after suturing the conjunctival–scleral wound, Meloxicam 0.2 mg/kg (Sub-Q) was administered in each eye along with 2 drops of admixed neomycin sulfate (3.5 mg/mL)-polymyxin B (10,000 units/mL)-dexamethasone (1 mg/mL). In addition to standard animal care, each animal was given one drop of admixed neomycin sulfate (3.5 mg/mL)-polymyxin B (10,000 units/mL)-dexamethasone (1 mg/mL) eye drops to each eye twice daily for the first five post-surgical days.

*Euthanasia*: On Day 1, Day 3, and Day 7, four rabbits were euthanized, and on Day 14, Day 28, and Day 56, six rabbits were euthanized by administration of sodium pentobarbital (150–200 mg/kg) IV.

*Procurement of Ocular Specimens:* Both eyes of each animal were removed immediately after euthanasia at each specified time-point. The eye globes were inspected to evaluate the insertion site of the micro-implant and observe any evident abnormalities of that site. For pharmacokinetic evaluation of the MTX concentration in the vitreous, the vitreous (~1 mL) was extracted manually from the eyes of the rabbits designated for pharmacokinetic evaluation and collected in a sterile transport vial. The vials containing the vitreous specimens were stored in dry ice and then kept frozen until the date of pharmacokinetic testing (see Pharmacokinetics Study below). The eye globes of the animals designated for histopathological evaluation were immersed immediately in 10% neutral buffered formalin for subsequent pathological processing and histopathological analysis (see Histopathological Study below).

*Pharmacokinetics Study:* The concentration of MTX in the vitreous samples obtained from the eyes receiving the MTX micro-implant and the placebo micro-implant for each time-point was analyzed using high-performance liquid chromatography (HPLC). The HPLC method was carried out as described in the United States Pharmacopeia assay for MTX [33]. This method has been previously described in our previous publication on the in vivo study of a similar PLA-coated CS-MTX micro-implant [11]. Briefly, the Agilent^®^ 1100 HPLC system (Agilent Technologies, Santa Clara, C, USAA) with a diode array detector was used for the HPLC analysis. A C-18 column measuring 150 mm × 4.6 mm with a pore size of 80 Å was used. The column temperature was set at 23 °C. Acetonitrile and phosphate/citrate buffer (pH 6.0) mixed in the ratio of 10:90 was used as the mobile phase. A flow rate of 1 mL/min of the mobile phase was used with an injection volume of 10 µL. The characteristic UV wavelength of 302 nm was used for the detection of MTX. For each time-point, (a) n = 3 vitreous samples obtained from the eye receiving the MTX micro-implant and (b) n = 3 vitreous samples obtained from the eye receiving the placebo micro-implant were analyzed.

*Histopathological Study:* Similar to our previous study, the globes were grossed and sectioned to display the micro-implant and surgical wound in pupil–optic nerve (P-ON) sections [11]. The globes were then processed and stained as previously described [11]. The stained histopathology slides were then evaluated by one of the authors (Z.M.C.) to evaluate any potential toxicity or complications.

## 3. Results

**Figure 3:**
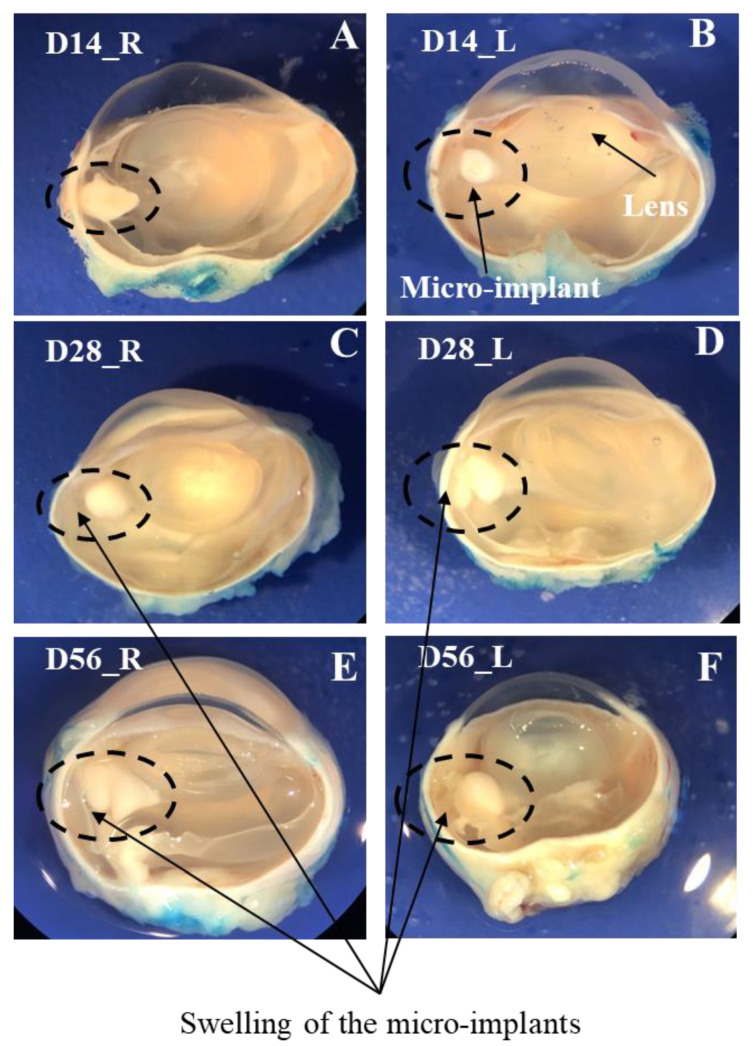
The position of the micro-implant relative to the lens can be seen in all the eye globes. The vitreous is clear in all the eye globes, indicating no toxicity caused by the MTX micro-implant and placebo micro-implant on the 14th day ((**A**,**B**), respectively); on the 28th day ((**C**,**D**), respectively); and on the 56th day ((**E**,**F**), respectively). Note the swelling of the micro-implants on the Day 28 and Day 56 time-points. Nomenclature of the eye globes—Dxx_y, where xx = time-points (14, 28, 56), and y = eye (R—right eye receiving the MTX micro-implant; L—left eye receiving the placebo micro-implant).

There were no complications during the surgical insertion of the micro-implants. The gross inspection of the position of the micro-implant relative to the crystalline lens and the eye wall incision showed (a) there was no migration of the micro-implants from their respective initial implantation site; (b) the gross appearance of the micro-implant and the vitreous surrounding the micro-implant was not appreciably different in the eyes that received an MTX-containing micro-implant and those that received a placebo micro-implant; and (c) there were signs of swelling of the micro-implant in the eyes on the Day 28 and Day 56 time-points (Figure 3).

**Figure 4:**
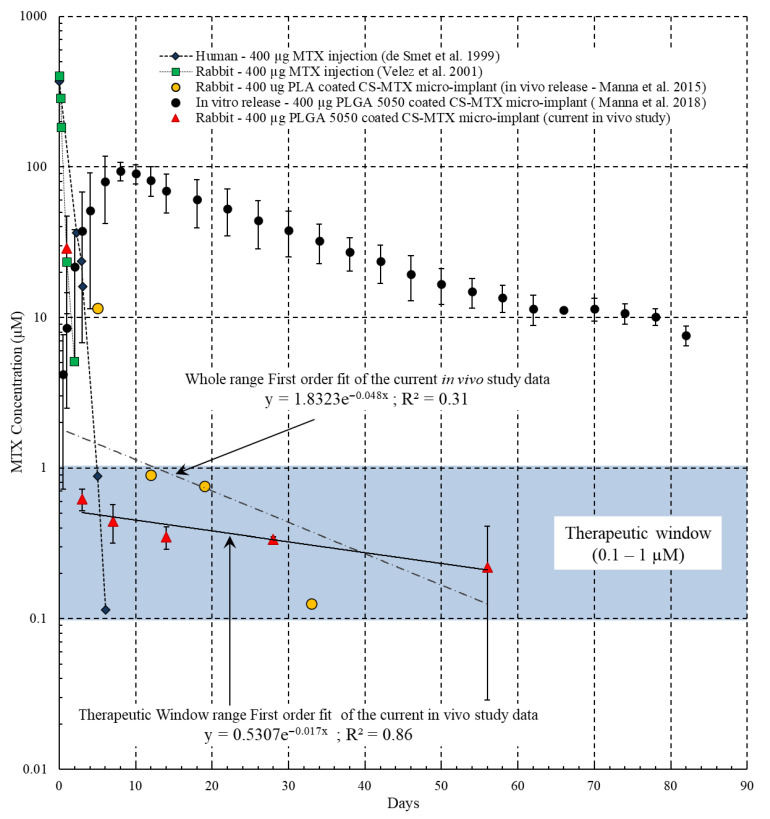
Comparison of concentration of MTX in the vitreous (therapeutic window—shaded region).

*Pharmacokinetics Study:* The concentration profile of MTX in the vitreous of the eye post-implantation on Days: 1, 3, 7, 14 and 56 is shown in Figure 4. The drug concentration is observed to be within the therapeutic window (0.1–1 µM) from Day 3 to Day 56. No MTX concentration was detected by the HPLC in the vitreous samples obtained from the eyes containing the placebo micro-implants. The MTX concentration data of our study have been compared with prior clinical study results [34] and the pre-clinical study results [15] obtained from a 400 µg MTX intravitreal injection and also with our prior in vitro study involving the same micro-implant as used in this study [32] (Figure 4).

A peak MTX concentration (Cmax-MTX) of 360 µM and 400 µM was observed from the clinical study [34] and the pre-clinical study [15], respectively, and the total duration of MTX release lasted for 2–6 days. In comparison, the MTX release profile observed in this current in vivo study showed a Cmax-MTX of 28.88 µM on the first day and subsequent delivery of MTX in the therapeutic window (0.1–1 µM) for about 2 months (56 days). The MTX concentration, obtained from our prior in vitro study [32] involving the same micro-implant, is comparatively higher than that of the MTX concentration observed in this in vivo study. The higher concentration of MTX in the in vitro study can be potentially attributed to the lack of realistic representation of the physiological convective–diffusive transfer of the vitreous in the in vitro environment. Additionally, phosphate-buffered saline (PBS) of pH 7.4 at ~38 °C used as the dissolution media in the in vitro study may not accurately replicate the solubility, diffusivity and convective clearance of MTX in the in vivo vitreous.

A regression value of R^2^~0.86 is obtained by fitting the in vivo vitreous concentration of MTX in the therapeutic release phase to the characteristic first-order equation model. The regression value worsens (R^2^~0.31) by fitting the in vivo vitreous concentration of MTX of the whole duration of release to the characteristic first-order equation model. This indicates the release mechanism of MTX in the therapeutic release phase follows first-order kinetics. Similarly, the half-life (t_1/2_) of MTX is 40.76 days, as obtained from the first-order fit (first-order constant *k* = 0.017) in the therapeutic release phase. The t_1/2_ of MTX worsens to ~14.43 days, as obtained from the first-order fit (first-order constant *k* = 0.048*)* of the whole duration of release.

**Figure 5:**
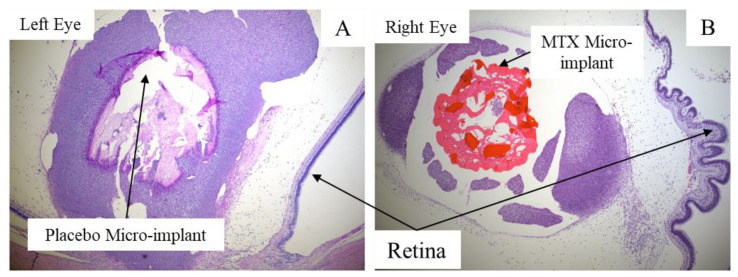
Histopathology findings using H&E staining. (**A**) microslide (4× shows the placebo micro-implant (Day 28) surrounded by a more pronounced inflammatory capsule; (**B**) microslide (4×) shows the MTX micro-implant (Day 28) with mild non-granulomatous inflammatory infiltrate surrounding the micro-implant and localized vitreous traction causing wrinkling of the retina. The retina appears anatomically healthy.

*Histopathology Study:* The details of the histopathological findings have been reported in the non-invasive study of toxicity and performance of the same micro-implant on the same rabbits, as used in this study [31]. Briefly, the issues identified in the histopathology analysis were associated with surgical procedures. Otherwise, the micro-implant showed no signs of toxicity and appeared to be safe for application in the VR domain. Histopathology findings include focal vitreoretinal traction without any apparent predominance between MTX and placebo micro-implants (Figure 5). The retina appeared to be anatomically healthy in all the eyes.

## 4. Discussion

Oral administration of MTX is used to treat uveitis (typical dose of 7–25 mg once weekly), often with supplemental oral folic acid to reduce systemic toxicity [7]. Additionally, administration of a single intravitreal MTX injection (typical dose of 200 to 400 µg/0.1 mL) or a series of injections over a several-month period has been used as an off-label treatment for severe or recalcitrant uveitis [8]. In selected cases of PIOL, intravitreal MTX (usually at the 400 µg/0.1 mL dose) [35] has been administered off-label to one or both eyes in addition to systemic MTX-based chemotherapy. Additionally, in patients without evidence of active brain–cerebrospinal fluid involvement, the intravitreal MTX injections are administered to the affected eye(s) alone [3].

This development of a sustained-release MTX intravitreal micro-implant stems from our motivation to create an alternative clinical approach to the current treatment modality of intravitreal MTX injections to treat VR diseases such as PIOL, uveitis and to prevent PVR. The FDA-approved sustained-release intraocular implants only deliver hydrophobic drugs (e.g., corticosteroids, ganciclovir, dexamethasone, fluocinolone acetonide) [17]. In comparison, this micro-implant platform offers a major advantage of delivering a sustained therapeutic concentration of hydrophilic drugs with a short half-life, such as MTX, over a prolonged duration. This study demonstrated the PLGA-coated CS-MTX micro-implants were able to deliver a therapeutic concentration of MTX (0.1–1 µM) over a period of 56 days in normal rabbit eyes without any signs of potential toxicity.

The PLGA-coated CS-MTX micro-implant showed a peak MTX concentration (Cmax-MTX) of 28.88 µM in the vitreous on the first-day time-point. This Cmax-MTX obtained from the PLGA-coated CS-MTX micro-implant is ~92% lower than the Cmax-MTX observed in intravitreal MTX injections (360–400 µM) [34]. Although the Cmax-MTX of 28.88 µM is beyond the therapeutic window (0.1–1 µM), it is anticipated to have an insignificant toxic effect, as it is still ~92% less than the Cmax-MTX of the intravitreal injections. Furthermore, the PLGA-coated CS-MTX micro-implant releases the same quantity of MTX (400 µg) over a duration of 56 days as compared to clearing after 2–3 days after intravitreal injection. Therefore, it can be expected that the PLGA-coated CS-MTX micro-implant will be well tolerated in the VR domain.

The t_1/2_ of our MTX micro-implant, as obtained from the first-order fit of the whole duration of release, is ~14.43 days. This is very similar to the t_1/2_ of MTX (13.1 days), as observed in the same PLGA-coated CS-MTX micro-implant in the in vitro study [32]. Additionally, the t_1/2_ of our MTX micro-implant is prolonged to 40.76 days when the first-order fit is applied to the concentration data points in the therapeutic release phase (excluding the Cmax-MTX on Day 1). Therefore, it is likely that the PLGA coating further extends the duration of release in addition to reducing the peak MTX vitreous concentration (Cmax).

In our previous in vivo study on the PLA-coated CS-MTX micro-implant (400 µg of MTX), a therapeutic concentration of MTX (0.1–1 µM) was observed for 33 days without any signs of toxicity in rabbit eyes (Figure 4). Based on (a) our experience of limited time-points from that study (Days: 5, 12, 19 and 33) [11] and (b) the in vitro release profile of the MTX and swelling profile of the PLGA 5050-coated CS-MTX micro-implant [32], we introduced more time-points in this study (Days: 1, 3, 7 14, 28 and 56) for improved knowledge of the drug release profile in the intravitreal domain. Additionally, due to prior experience from the in vivo study involving the PLA-coated CS-MTX micro-implant, histopathology was conducted only at later time-points (Days 14, 28 and 33), as insignificant changes due to either toxicity or inflammation in the intraocular domain were expected at early time-points (Days: 1, 3 and 7).

The swelling of the micro-implants, as observed in cross-sections of the eye globes (Figure 3), on Day 28 and Day 56 is consistent with the findings of the in vitro study [32] and the findings of the SLB, fundoscopy and US in the non-invasive in vivo study on the same micro-implant [31]. Additionally, in the in vitro study [32], the PLGA-coated micro-implant showed peak swelling (6.2 times its original weight) around 30 days, which is similar to swelling observed on Day 28 of this study, and finally disintegrated around 66 days in vitro, which is approximately the duration of this study (~56 days). Furthermore, the ultrasound study also showed that (a) none of the micro-implants migrated in the vitreous; (b) the overall shape of each of the micro-implants was stable during the study barring the swelling towards the end of the experiment; and (c) fully attached peripheral retina and no evident abnormalities of the peripheral vitreous adjacent to the micro-implant in any of the evaluated eyes.

This study did not manifest any evidence of histological retinal deterioration at any postsurgical time-point related either to the PLGA-coated CS-MTX micro-implants or to the placebo micro-implants. This corroborates the findings of the electroretinogram (ERG) evaluation on the same rabbits across the duration of this study [31]. Statistical evaluation of the ERG analysis showed there was no change in the retinal functional integrity due to the micro-implants over the entire duration of the study.

Lastly, the histopathological evaluation corroborated the absence of toxicity such as thinning of the inner retina, photoreceptor atrophy and vascular events at later time-points [31], indicating observed changes are associated specifically with the implantation procedure and not caused by any toxicity associated with the micro-implants. Additionally, the materials used to fabricate the micro-implant are considered to be biodegradable. PLGA is known to be metabolized under physiological conditions to produce carbon dioxide and water, and CS is expected to be degraded by lysozyme, an enzyme present in the vitreous to form amino sugars [36].

While this study provides an improved evaluation of the pharmacokinetics and toxicity performance of this micro-implant, further studies are required in the future such as (a) cell invasion evaluation at later time-points; (b) assessment of MTX concentration in the aqueous humor; (c) improved sample size; (d) inclusion of more time-points for an improved resolution of the release concentration; and (e) immunohistochemistry.

## 5. Conclusions

Our investigation showed that the PLGA-coated CS-MTX micro-implant delivered a therapeutic concentration of MTX in rabbit eyes for ~56 days. Histopathology analysis demonstrated that the micro-implant appeared safe and well tolerated in the rabbit eyes. Our PLGA-coated CS-MTX micro-implant may serve as a potential platform for sustained release of a therapeutic concentration of MTX or other similar hydrophilic drugs in the VR domain. Additionally, it can be used as a promising alternative to the current treatment modality of recurrent intravitreal MTX injections, thereby avoiding the complications, discomfort and treatment burden associated with the intravitreal MTX injections.

## Figures and Tables

**Table 1:** Design of the minimally invasive study.

Time-point	Number of Rabbits	Pharmacokinetic Evaluation	Gross Inspection of the Cross-Section of the Eye Globe	Histopathology ^
Day 1	3 + 1 (control *)	3 rabbits	None	None
Day 3	3 + 1 (control)	3 rabbits	None	None
Day 7	3 + 1 (control)	3 rabbits	None	None
Day 14	5 + 1 (control)	3 rabbits	2 rabbits	2 rabbits
Day 28	5 + 1 (control)	3 rabbits	2 rabbits	2 rabbits
Day 56	5 + 1 (control)	3 rabbits	2 rabbits	2 rabbits

Nomenclature: * For control rabbits, no pharmacokinetic evaluation, gross inspection of the eye globe and histopathology were conducted; ^ No pharmacokinetic evaluation was conducted in the eyes used for histopathology evaluation.

## Data Availability

The data presented in this study are available on request from the corresponding author.
